# Associations between Depression, Nutritional Status and Mediterranean Diet in Dalmatian Kidney Transplant Recipients

**DOI:** 10.3390/nu13124479

**Published:** 2021-12-15

**Authors:** Marijana Vučković, Josipa Radić, Andrea Gelemanović, Dora Bučan Nenadić, Ela Kolak, Mislav Radić

**Affiliations:** 1 Department of Nephrology and Dialysis, University Hospital Centre Split, 21000 Split, Croatia; mavuckovic@kbsplit.hr; 2 Department of Internal Medicine, University of Split School of Medicine, 21000 Split, Croatia; mislavradic@gmail.com; 3 Mediterranean Institute for Life Sciences (MedILS), 21000 Split, Croatia; andrea.gelemanovic@gmail.com; 4 Department of Nutrition and Dietetics, University Hospital Centre Split, 21000 Split, Croatia; dorabucan@gmail.com (D.B.N.); elakolak93@gmail.com (E.K.); 5 Department of Clinical Immunology and Rheumatology, University Hospital Centre Split, 21000 Split, Croatia

**Keywords:** depression, nutrition, nutritional status, Mediterranean diet, kidney transplant, Dalmatia

## Abstract

Depression has been addressed as a predictor of worse outcomes in kidney transplant recipients (KTRs). Nutritional status plays a great role in treatment of this population. The Mediterranean diet (MeDi) has been associated with lower levels of depressive symptoms. The aim of this cross-sectional study was to determine the rate of depression and its correlations to nutritional status and dietary habits according to the MeDi in Dalmatian KTRs. We included 115 KTRs, and data about body composition and anthropometric, laboratory and clinical parameters were obtained for each study participant. The Beck Depression Inventory-II (BDI-II) questionnaire was used to assess depressive symptoms and the Mediterranean Diet Serving Score (MDSS) was used to assess adherence to the MeDi. We found the presence of depressive symptoms in 21.73% of the Dalmatian KTRs. BDI-II score was reciprocally associated with fat mass, trunk visceral fat, anthropometric parameters of obesity, triglyceride levels and olive oil consumption. Inverse associations were found between BDI-II score and skeletal muscle mass, handgrip strength, MCV, hemoglobin levels and consumption of fish and white meat, as suggested by the MeDi. Our results showed the interconnections between nutritional status, dietary habits and depression in Dalmatian KTRs.

## 1. Introduction

Kidney transplantation (KTX) results in better kidney function and a great deal of metabolic [1], lifestyle and psychological changes [2]. There are high rates of depression among patients suffering from chronic kidney disease (CKD) and, although reduced after KTX [3], rates of depression remain higher than in the general population [4]. Recent studies have linked depression with worse outcomes in kidney transplant recipients (KTRs), with a special focus on overall mortality [5,6], glomerular filtration rate (eGFR) [6,7], malnutrition [8], cardiovascular risk [5] and treatment non-adherence [9].

Additional important factors in the KTR population are nutritional status [10,11] and cardiometabolic status [12], which are also linked with multiple adverse outcomes. Weight gain is common after KTX and it has a multifactorial etiology [13]—from corticosteroid induced hyperphagia, the cessation of dietary restrictions, a lack of physical activity, psychological factors and a lack of information on healthy dietary patterns [10]. In the KTR population, obesity has been associated with higher mortality, cardiovascular risk [14], metabolic syndrome, type 2 diabetes mellitus (T2DM) [15] and graft failure [15].

In the general population, a bidirectional relationship between obesity and depression was found. A sedentary lifestyle, poor diet, microbiome, genetics and inflammation are only some of the factors believed to contribute to the aforementioned relationship [16]. Data about the relationship between obesity and depression in KTRs are still lacking. The results of a prospective study on KTRs point to an association between weight change and the level of depressive symptoms at 6 months after KTX [17]. Studies on other chronic noncommunicable diseases, such as coronary artery disease, have stated that there is a correlation between depression and nutritional status [18].

A Mediterranean lifestyle, implying adherence to the Mediterranean diet (MeDi) and high levels of socializing and physical activity in a healthy population, has been indicated as a protective factor for depression [19]. In a recent RCT, the MeDi has been associated with the improvement of depression and mental health in the general population [20]. In another study, an association between lower adherence to the MeDi and more depressive symptoms was found [21]. In KTRs, the MeDi has been found to be a protective factor for graft function decline [22], development of new-onset diabetes mellitus [23] and metabolic syndrome [24]. Our previous research showed associations between adherence to the MeDi and higher muscle mass and associations between particular components of the MeDi and body mass index (BMI), fat mass, serum lipid levels and waist-to height ratio (WHtR).

Since mental health care and nutritional care are not a part of the routine post-transplantation care of our center, the aim of this study was to determine the rate of depression and its correlations to nutritional status and dietary habits according to the MeDi in KTRs in the Mediterranean region, Dalmatia, Croatia.

## 2. Materials and Methods

This study was conducted using the protocol described in our previously published study [25], where the methods have been described in detail.

### 2.1. Study Design and Population

We included 159 kidney transplant recipients (KTRs) in this cross-sectional study at the outpatient clinic of the Department of Nephrology and Dialysis, University Hospital of Split, Croatia, between July 2019 and October 2019. The study protocol was approved by the Ethics Committee of the University Hospital of Split, Croatia.

We excluded patients who met one of the following exclusion criteria: had an implanted pacemaker or cardioverter defibrillator, stents, or limb amputation; refused to participate in the study; did not fill out the whole Beck Depression Inventory-II (BDI-II) questionnaire; had an active infection; or had active malignant disease.

### 2.2. Body Composition and Anthropometric Measurement

An MC-780 Multi Frequency Segmental Body Analyzer (Tanita, Tokyo, Japan) was used to assess body composition using bioelectrical impedance analysis (BIA) technology for each study participant.

Regarding anthropometric parameters, we obtained data about height, weight, body mass index (BMI), waist circumference (WC), mid-upper arm circumference (MUAC) and the waist-to-height ratio (WHtR).

Handgrip strength was assessed using a hand-held dynamometer (Saehan, Korea) three times and an average value was calculated.

### 2.3. Depression Assessment

The Beck Depression Inventory-II (BDI-II) self-administered questionnaire was used to assess the severity of depressive symptoms. The questionnaire consists of 21 questions and answers range from 0–3 where 3 indicates more severe symptoms of depression. The maximum score is 63, and cut-offs were determined as follows: 0–13 as minimal depression, 14–19 as mild depression, 20–28 as moderate depression and 29–63 as severe depression. Due to the low number of KTRs in the groups of moderate and severe depression, a binary variable was created and a cut-off of ≥14 depicted depression.

### 2.4. Mediterranean Diet Serving Score

To assess adherence to the Mediterranean diet (MeDi), we used a validated Mediterranean Diet Serving Score (MDSS) questionnaire which considers the consumption of 14 different foods and food groups in time intervals per meal, day or week. The maximum score is 24 and the optimal cut-off point to determine adherence to the MeDi is ≥13.50 [26].

### 2.5. Medical History and Clinical and Laboratory Parameters

A thorough examination of medical records, analysis of blood samples and 24 h urine samples was performed as previously described [25].

### 2.6. Statistical Analysis

Categorical data were described with numbers and percentages, while numerical data were described with means and standard deviations (SD) in cases of parametric distributions and with medians and interquartile ranges (IQR) in cases of nonparametric distributions. Normality was assessed using the Shapiro–Wilk test. To examine the differences between the two groups (KTRs with and without depressive symptoms), chi-square tests were used for categorical data, *t*-tests were used for parametric numerical data and Mann–Whitney *U* tests were used for nonparametric numerical data. Spearman’s rank correlation was used to assess the association between BDI-II score and the measured parameters. To find predictors for depression in KTRs, first, a logistic regression analysis was performed, adjusted for age, sex and eGFR (model 1). Then, all statistically significant variables from descriptive statistics, correlations and regression analyses were used as inputs to the Boruta algorithm [27], a random forest classification algorithm, which selected the most relevant variables for depression. The Boruta algorithm iteratively compared the importance of features by comparing them with the importance of their random shuffled copies called shadow features. After a maximum of 1000 iterations, where shadow features were recreated at each iteration, the algorithm selected the most important features that should be kept. If some features were left without a definite decision, they were also used in the follow-up step. The selected variables were checked for collinearity and inappropriate ones (defined as having a variance inflation factor (VIF) > 4) were removed, and the remaining variables were used as independent variables in the multivariate logistic regression model (model 2). Finally, output from model 2 was used in a stepwise logistic regression model with both forward and backward selection to identify the most important predictors for depression among KTRs. Models 2 and 3 were compared with the Akaike information criterion (AIC) and the quality of the models was evaluated with the Hosmer and Lemeshow goodness of fit test. The results of logistic regressions were given as odds ratios (OR) with 95% confidence intervals (CI). The significance level was set at a *p*-value of <0.05. All statistical analyses were performed using the free software environment for statistical computing, R version 4.0.0 [28].

## 3. Results

This study included 115 KTR patients. Data about general parameters, medical history, anthropometric and body composition parameters, laboratory parameters, MDSS score and the presence of depressive symptoms are shown in Table 1. When applying the recommended cut-off for BDI-II score, 15 (13.04%), 6 (5.22%) and 4 (3.48%) KTRs showed the presence of mild, moderate and severe depressive symptoms, respectively. Due to the low numbers, we applied a cut-off point of ≥14 for BDI-II score for the presence of any depressive symptoms. Our results showed that the median BDI-II score of Dalmatian KTRs was 8 (IQR = 10) and that 25 (21.74%) of them showed depressive symptoms (median BDI-II score of 18, IQR = 11). The mean MDSS score was 10.45 (SD = 4.27), and only 27 (23.48%) scored 14 or more, meaning they were adherent to the MeDi recommendations. Statistically significant differences in the measured parameters between the groups with and without depressive symptoms were found. KTRs without depressive symptoms were younger, had higher values of mean cellular volume (MCV) and higher values of skeletal muscle mass in comparison to those KTRs with depressive symptoms. No other differences between the groups were found (Table 1).

**Figure 1:**
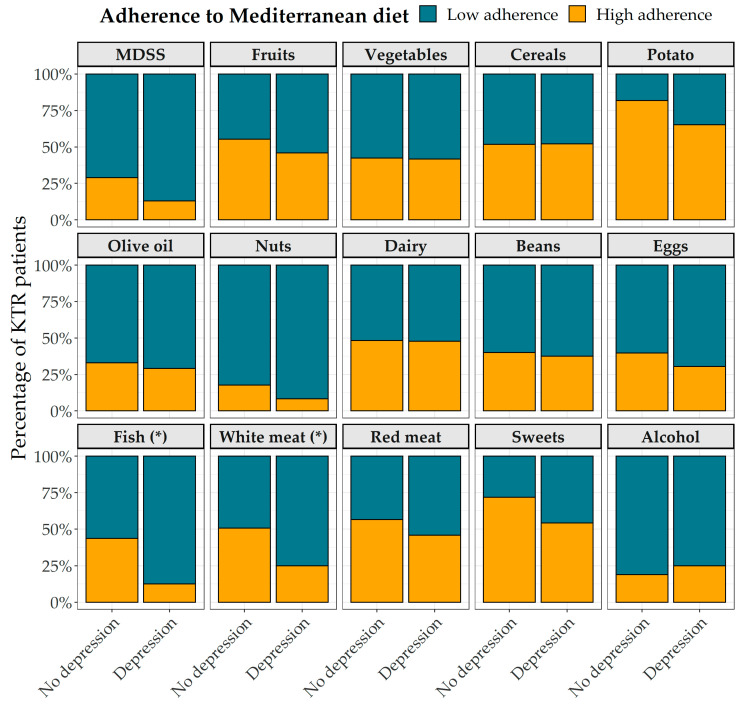
Adherence to the Mediterranean diet according to the presence of depressive symptoms among Dalmatian KTRs. Statistical significance (tested with chi-square tests) was found only for adherence to the fish (* *p* = 0.011) and white meat (* *p* = 0.046) recommendations. Abbreviations: MDSS, Mediterranean Diet Serving Score; KTR, kidney transplant recipient.

Regarding adherence to the MeDi and differences according to the presence of depressive symptoms, no statistically significant differences in total MDSS were found. Nevertheless, KTRs without depressive symptoms were more prone to follow suggestions of the MeDi for fish and white meat. Adherence to the MeDi and its separate components for the whole studied population is shown in Figure 1.

Correlations between the BDI-II score and the measured parameters are shown in Table 2 (only the statistically significant parameters are shown). Negative correlations were found between the BDI-II score and handgrip strength, the percentage of skeletal muscle mass, the levels of mean cellular volume (MCV) and hemoglobin (Hb). Positive correlations between the BDI-II score and age and the percentage of fat mass were found. There was also a borderline significance for a positive correlation between the BDI-II score and adherence to the recommendations of the MeDi on olive oil intake.

In addition, significant correlations between BMI and the percentage of fat mass and trunk visceral fat were found (r = 0.65, *p* < 0.001), (r = 0.82, *p* < 0.001), respectively.

Statistically significant predictors for the presence of depressive symptoms by multivariate logistic regression (model 1; adjusted for age, sex and eGFR) are shown in Table 3. The presence of depressive symptoms in Dalmatian KTRs was associated with higher weight, waist circumference, fat mass, BMI, trunk visceral fat, MUAC and levels of triglycerides.

**Figure 2:**
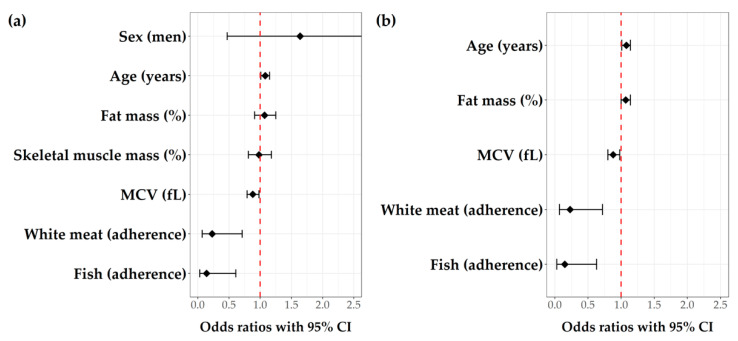
Multivariate logistic regression analyses for the presence of depressive symptoms among Dalmatian KTRs. (**a**) Model 2, after the Boruta feature selection algorithm and removal of parameters with high collinearity (AIC = 103.15, Nagelkerke R^2^ = 0.387, Hosmer and Lemeshow goodness of fit test *p*-value = 0.939); (**b**) Model 3, the final model, after stepwise logistic regression with both forward and backward selection (AIC = 99.81, Nagelkerke R^2^ = 0.380, Hosmer and Lemeshow goodness of fit test *p*-value = 0.852). Abbreviations: MCV, mean cellular volume (fL).

After applying the Boruta algorithm as a feature selection method and removing parameters that showed large collinearity (results not shown), sex, age, percentage of fat mass and skeletal muscle mass, mean cellular volume (MCV) and adherence to fish and white meat were selected as most relevant variables to affect the presence of depressive symptoms and were used in a multivariate logistic regression (model 2, Figure 2a). Following that, a stepwise logistic regression model with both forward and backward selection was applied (model 3, Figure 2b). The final model showed that the most important predictors for depressive symptoms among Dalmatian KTRs are older age (OR (95% CI) = 1.08 (1.01–1.14), *p* = 0.020), higher percentage of fat mass (OR (95% CI) = 1.07 (1.00–1.14), *p* = 0.049), lower mean cellular volume (OR (95% CI) = 0.88 (0.80–0.98), *p* = 0.017) and no adherence to fish (OR (95% CI) = 0.15 (0.03–0.63), *p* = 0.010) and white meat (OR (95% CI) = 0.23 (0.07–0.72), *p* = 0.012) based on the MeDi recommendations.

**Figure 3:**
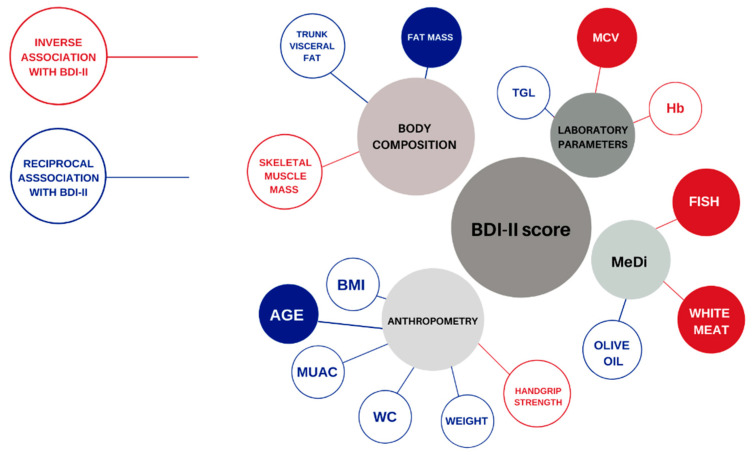
Graphical summary of the inverse and reciprocal associations of the measured parameters with the presence of depressive symptoms among Dalmatian KTRs. Abbreviations: BDI-II, Beck Depression Inventory-II score; BMI, Body Mass Index (kg/m^2^); MUAC, mid-upper arm circumference (cm); WC, waist circumference (cm); Hb, hemoglobin (g/L); MCV, mean cellular volume (fL); Tgl, triglycerides (mmol/L). Inverse associations with BDI-II score are depicted in red, and reciprocal associations are depicted in blue. The most important predictors for depressive symptoms among Dalmatian KTRs are highlighted with fully colored circles.

A graphical summary of all of the results within this study is shown in Figure 3.

## 4. Discussion

To our knowledge, this is the first study in this region that evaluated the associations between depression, nutritional status and dietary habits in KTRs.

The prevalence of depression in Dalmatian KTRs (21.73%) was similar to other studies within the same population where it was reported to be between 20% and 25% [4,7,29]. Our results showed associations between older age and a higher number of depressive symptoms which is similar to previous research [17].

### 4.1. Body Composition and Depression in Dalmatian KTRs

Depression has been associated with lower muscle mass in the general population [30], similar to our findings in KTRs. Previous studies did not evaluate the association between depression and body mass composition in this patient population. Our results also showed associations between more depressive symptoms and lower muscle strength. As muscle mass and muscle strength are determinants of sarcopenia, we could state the correlation between depression and sarcopenia in Dalmatian KTRs. The study from Kurita et al. found depression and hopelessness to be predictors of sarcopenia in CKD and hemodialysis patients [31].

Furthermore, positive associations between trunk visceral fat and fat mass with BDI-II score were shown in our results. Adipose tissue and its secretory function have been linked to inflammatory processes which are believed to participate as mediators in the binary relation between adiposity and depression [32,33].

### 4.2. Anthropometric Parameters and Depression in Dalmatian KTRs

The positive associations between BDI-II scores and BMI found in our study are opposite to the results from Nohre et al. who found no association between BMI and depression in KTRs [34]. A possible explanation for our finding is that the higher BMI in our study population reflects the higher content of fat mass.

Additionally, the positive associations between BDI-II scores and waist circumference and MUAC speak in favor of obesity and depression being related in Dalmatian KTRs.

### 4.3. Mediterranean Diet and Depression in Dalmatian KTRs

When discussing adherence to the Mediterranean diet, we found no difference in total MDSS between depressive and non-depressive KTRs, implying a poor dietary pattern in both groups (average MDSS score being 10.45, which is lower than the recommended threshold of ≥13.50 for good adherence).

Interestingly, our results suggest that KTRs without depressive symptoms adhere more to the recommendations of the MeDi on fish and white meat intake, and these two components of the MeDi were also selected as one of the most important predictors for depression. A possible explanation for this finding could be the influence of omega-3 fatty acids, which are known to be associated with a lower risk for depression [35,36,37]. The results of a recent study state that there is a beneficial impact of white meat on depressive symptoms in the general population, while there is a negative impact of red meat intake [38]. In a recent systematic review, a higher risk or prevalence of depression has been linked to meat avoidance [39]. Another factor correlated to white meat and fish and related to fewer depressive symptoms could be that the protein load of the white meat and fish could add to sarcopenia prevention.

The borderline-significant positive correlation between olive oil consumption and depressive symptoms could be due to the low number of Dalmatian KTRs adhering to the olive oil recommendations by the MeDi.

### 4.4. Laboratory Parameters and Depression in Dalmatian KTRs

A negative correlation between BDI-II scores and hemoglobin levels in Dalmatian KTRs was found in our study and was also observed by Zelle and Czira [5,8]. Furthermore, we found a negative association between BDI-II scores and MCV levels, which could imply that the iron deficiency [40] is also contributing to the level of depressive symptoms, as described in previous research on the general population.

Positive associations between triglycerides and BDI-II scores in Dalmatian KTRs are similar to the results in the general population [41]. This finding can be explained by the poor dietary pattern which contributes to the triglyceride level.

A recent study has suggested promotion of self-efficacy and self-care behaviors for depressive symptoms of KTRs [42]. One of the self-care behaviors could be in maintaining healthy dietary habits and lifestyle changes in order to reduce body weight and change the body composition by enhancing muscle mass and reducing fat mass.

Previous research showed the complex interconnections between depression, muscle strength, trunk visceral fat and fat mass in the general population [43,44]. Associations between the MeDi and lower depression rates [19], as well as red meat consumption and the risk of depression are known from previous research in the general population [38]. The novelty of our research is found in this specific target population of KTRs and the interconnections between depressive symptoms, parameters of body mass composition and components of the MeDi in this population of patients. Physical activity levels in KTRs should not be overlooked when it comes to depression treatment [45], but we did not take it into consideration for this study. Another limitation of our study is the small number of KTRs with depressive symptoms. Assessment of adherence to the MeDi using a one-time questionnaire, although a widely used and validated tool, could potentially influence the results by over or underestimation of food intake.

Additional limitations of this study mainly arise from its cross-sectional design which is preventing any causative relations. It is also a single-center study but with a representative study population, due to the great number of people gravitating to our center. A further limitation of this study is that we did not take into consideration data about other factors that influence body composition, such as exercise and daily calorie and protein intake.

## 5. Conclusions

Our results emphasize the interconnection of mental health, nutritional status and dietary habits of Dalmatian KTRs. Mental health, together with nutritional care, should not be overlooked when treating patients in the post-transplantation phase. More prospective studies are needed in order to determine a causal relation between depression, nutritional status and the MeDi in KTRs.

## Figures and Tables

**Table 1:** Basic characteristics and differences regarding the presence of depressive symptoms among Dalmatian KTRs.

	Total (*N* = 115)	No Depression (*N* = 90)	Depression (*N* = 25)	*p* *
BDI-II score, median (IQR)	8 (10)	6 (7)	18 (11)	**<0.001**
Age (years), median (IQR)	60 (16)	60 (16)	67 (12)	**0.011**
Sex, N (%)				
Women	54 (46.96)	41 (45.56)	13 (52)	0.730
Men	61 (53.04)	49 (54.44)	12 (48)	
Time since transplantation (years), median (IQR)	5 (7)	5 (6.88)	7 (8.25)	0.190
Dialysis duration (years), median (IQR)	2 (3.5)	2 (3)	4 (4)	0.080
Dialysis type, N (%)				
PD	38 (33.63)	31 (35.23)	7 (28)	0.565
HD	67 (59.29)	50 (56.82)	17 (68)	
PD + HD	8 (7.08)	7 (7.95)	1 (4)	
Smoking status, N (%)				
Smoker	52 (50.98)	40 (51.28)	12 (50)	0.874
Former smoker	26 (25.49)	19 (24.36)	7 (29.17)	
Non-smoker	24 (23.53)	19 (24.36)	5 (20.83)	
COMORBIDITIES				
Presence of arterial hypertension, N (%)				
No	16 (13.91)	12 (13.33)	4 (16)	0.989
Yes	99 (86.09)	78 (86.67)	21 (84)	
Presence of diabetes mellitus, N (%)				
No	89 (77.39)	71 (78.89)	18 (72)	0.647
Yes	26 (22.61)	19 (21.11)	7 (28)	
Presence of chronic kidney disease, N (%)				
eGFR > 60 mL/min/1.73 m^2^	31 (28.44)	23 (26.74)	8 (34.78)	0.618
eGFR < 60 mL/min/1.73 m^2^	78 (71.56)	63 (73.26)	15 (65.22)	
ANTHROPOMETRIC PARAMETERS				
Height (cm), mean (SD)	172.68 (10.06)	173.1 (10.27)	171.2 (9.35)	0.407
Weight (kg), median (IQR)	78.51 (14.69)	79.16 (14.63)	76.21 (14.94)	0.378
BMI (kg/m^2^), mean (SD)	26.21 (4.1)	26.26 (4)	26.06 (4.51)	0.836
Middle upper arm circumference (cm), median (IQR)	29 (7)	28.5 (7)	30 (7.75)	0.888
Waist circumference (cm), mean (SD)	99.2 (12.54)	99.12 (12.51)	99.5 (12.95)	0.901
WHtR, mean (SD)	0.58 (0.07)	0.57 (0.07)	0.59 (0.08)	0.417
Handgrip strength (pounds)	40 (19)	42 (16.7)	34 (19.5)	0.134
LABORATORY PARAMETERS				
Alb (g/L), median (IQR)	42 (4)	42 (4.5)	41 (5)	0.082
Ca (mmol/L), median (IQR)	2.44 (0.18)	2.42 (0.2)	2.47 (0.11)	0.204
CRP (mg/L), median (IQR)	2.4 (4.38)	2.4 (4.3)	3.3 (4.6)	0.939
E, median (IQR)	4.65 (0.68)	4.61 (0.64)	4.77 (0.77)	0.485
GUP (mmol/L), median (IQR)	5.2 (1.1)	5.2 (1)	5.2 (1.2)	0.632
Hb (g/L), median (IQR)	134 (18)	135 (19.5)	133 (7.5)	0.537
K (mmol/L), mean (SD)	4.13 (0.47)	4.16 (0.47)	4.01 (0.49)	0.180
Total cholesterol (mmol/L), mean (SD)	5.98 (1.31)	5.97 (1.26)	6.04 (1.51)	0.824
Creatinine (mmol/L), median (IQR)	122 (55)	121 (54.5)	128 (57.5)	0.595
LDL (mmol/L), median (IQR)	3.62 (1.09)	3.63 (1.07)	3.58 (1.19)	0.866
MCV (fL), mean (SD)	87.67 (5.5)	88.29 (5.44)	85.38 (5.21)	**0.024**
Na (mmol/L), median (IQR)	141 (3)	141 (2.75)	141 (2.5)	0.342
P (mmol/L), median (IQR)	1.02 (0.23)	1.02 (0.23)	1.02 (0.27)	0.323
Tgl (mmol/L), median (IQR)	1.85 (1.48)	1.9 (1.45)	1.7 (1.4)	0.685
Uric acid (mmol/L), median (IQR)	392 (74.25)	391 (62)	394 (113)	0.977
Urea (mmol/L), median (IQR)	9.5 (4.78)	9.3 (4.33)	10.7 (7.67)	0.469
eGFR (ml/min/1.73 m^2^), median (IQR)	46.6 (26.6)	47.2 (22.85)	45.9 (33.35)	0.859
BODY COMPOSITION				
Fat mass (kg), median (IQR)	19.15 (10.57)	19.05 (10.25)	21.2 (10.1)	0.230
Fat mass (%), mean (SD)	23.68 (8.55)	23.04 (8.36)	25.94 (9.02)	0.143
Fat-free mass (kg), median (IQR)	59.3 (17.48)	59.45 (17.2)	54.9 (16.2)	0.107
Visceral fat, mean (SD)	9.17 (3.79)	8.83 (3.85)	10.43 (3.31)	0.070
Muscle mass (kg), median (IQR)	56.3 (17.05)	56.45 (17.38)	52.15 (15.48)	0.113
Skeletal muscle mass (kg), median (IQR)	31.25 (11.77)	32.1 (12.07)	28.25 (9.25)	**0.042**
Skeletal muscle mass (%), median (IQR)	40.9 (7.93)	42.3 (8.55)	38.4 (5.73)	**0.012**
Body mass (kg), median (IQR)	3 (0.8)	3 (0.77)	2.75 (0.73)	0.079
Phase angle, median (IQR)	5.15 (1.17)	5.2 (1.07)	4.85 (0.98)	0.178
Trunk visceral fat (kg), median (IQR)	10.2 (6.5)	9.9 (7.28)	10.6 (5.25)	0.212
Mediterranean Diet Serving Score (MDSS)				
Total MDSS points, mean (SD)	10.45 (4.27)	10.78 (4.35)	9.26 (3.84)	0.131
Adherence to MeDi, N (%)				
MDSS < 14 points	79 (74.53)	59 (71.08)	20 (86.96)	0.202
MDSS ≥ 14 points	27 (25.47)	24 (28.92)	3 (13.04)	

* *p*-values were obtained with chi-square tests for categorical data, *t*-tests for parametric numerical data and Mann–Whitney U tests for nonparametric numerical data. Bolded values represent statistically significant values. Abbreviations: BDI-II, Beck Depression Inventory-II score; PD, peritoneal dialysis; HD, hemodialysis; eGFR, estimated glomerular filtration rate using CKD-EPI (ml/min/1.73 m^2^); BMI, Body Mass Index (kg/m^2^); WHtR, waist-to-height ratio; Alb, serum albumin (g/L); Ca, calcium (mmol/L); CRP, C-reactive protein (mg/L); E, erythrocyte count; GUP, glucose (mmol/L); Hb, hemoglobin (g/L); K, potassium (mmol/l); LDL, low-density lipoprotein cholesterol (mmol/L); MCV, mean cellular volume (fL); Na, sodium (mmol/L); P, phosphates (mmol/L); Tgl, triglycerides (mmol/L); MDSS, Mediterranean Diet Serving Score.

**Table 2:** Statistically significant correlations between BDI-II score and measured parameters among Dalmatian KTRs.

Parameter	R	*p* *
Handgrip strength	−0.285	0.005
Skeletal muscle mass (%)	−0.278	0.003
MCV (fL)	−0.201	0.038
Hb (mmol/L)	−0.196	0.041
Olive oil (adherence)	0.189	0.049
Age (years)	0.210	0.024
Fat mass (%)	0.240	0.012

* *p*-values were obtained with Spearman’s rank correlation. Abbreviations: r, Spearman’s rank correlation coefficient; MCV, mean cellular volume (fL); Hb, hemoglobin (mmol/L).

**Table 3:** Multivariate logistic regression analyses for the presence of depressive symptoms among Dalmatian KTRs (adjusted for age, sex and eGFR; model 1; only statistically significant associations shown).

Predictor	OR	95% CI	*p*	Nagelkerke R^2^
Weight (kg)	1.08	1.01–1.16	0.033	0.16
Waist circumference (cm)	1.09	1.01–1.19	0.034	0.20
Fat mass (%)	1.12	1.01–1.24	0.038	0.13
Fat mass (kg)	1.15	1.02–1.30	0.022	0.18
BMI (kg/m^2^)	1.29	1.03–1.63	0.030	0.16
Trunk visceral fat	1.29	1.04–1.60	0.019	0.19
Middle upper arm circumference (cm)	1.38	1.05–1.80	0.019	0.26
Tgl (mmol/L)	5.83	1.02–33.48	0.048	0.32

Abbreviations: BMI, Body Mass Index (kg/m^2^); Tgl, triglycerides (mmol/L).

## Data Availability

Data may be requested from the author via e-mail: josiparadic1973@gmail.com.
